# *Vibrio cidicii* genomes recovered from Baltic Sea samples in Denmark

**DOI:** 10.1128/mra.01121-24

**Published:** 2024-12-12

**Authors:** Yaovi Mahuton Gildas Hounmanou, Beau-Gard Jules Hougbenou, Victorien Tamegnon Dougnon, Jens-Andre Hammerl, Anders Dalsgaard

**Affiliations:** 1 Department of Veterinary and Animal Sciences, Faculty of Health and Medical Sciences, University of Copenhagen, Frederiksberg, Denmark; 2 Research Unit in Applied Microbiology and Pharmacology of Natural Substances, Laboratory Research in Applied Biology, Polytechnic School of Abomey-Calavi, University of Abomey-Calavi, Abomey-Calavi, Benin; 3 DVG-Consultant Laboratory for Vibrio spp. in Food, Department Biological Safety, German Federal Institute for Risk Assessment, Berlin, Germany; University of Southern California, Los Angeles, California, USA

**Keywords:** *Vibrio*, Vibriosis, baltic

## Abstract

We report the genomic characteristics of the human pathogen *Vibrio cidicii* isolated from seawater and green algae in the Baltic Sea. Initially misidentified as *Vibrio vulnificus* through culture and MALDI-TOF, whole-genome sequencing (WGS) confirmed them as *V. cidicii*, highlighting the importance of WGS analysis in accurate classification of emerging pathogens.

## ANNOUNCEMENT

*Vibrio cidicii* is a Gram-negative, motile, rod-shaped bacterium, identified in 2016 from human blood and river water samples (1). It has pathogenic potential in humans (1, 2) and can cause gastroenteritis, wound infections, and septicemia and can be multidrug resistance (2–4).

Twelve samples of seawater and green algae (*Cladophora glomerata*) were collected from coastal areas of the Baltic Sea in Denmark during August 2023. Samples were enriched in alkaline peptone water (APW) for 6 h at 37°C (25 g of green algae and 25 mL of seawater in 225 mL of APW) and subcultured onto thiosulfate citrate bile salts sucrose agar (Oxoid Basingstoke, United Kingdom) and ChromAgar Vibrio (CHROMagar, Paris, France) and incubated for 18 h at 37°C. Presumptive *Vibrio vulnificus* colonies were selected from both agar types and streaked onto nutrient blood agar to ensure purity. Pure cultures were identified as *V. vulnificus* by MALDI-TOF (Bruker Daltonik GmbH, Bremen, Germany) using the Standard_FAMS and MBT-AutoX methods following the manufacturer’s guidelines.

**Fig 1:**
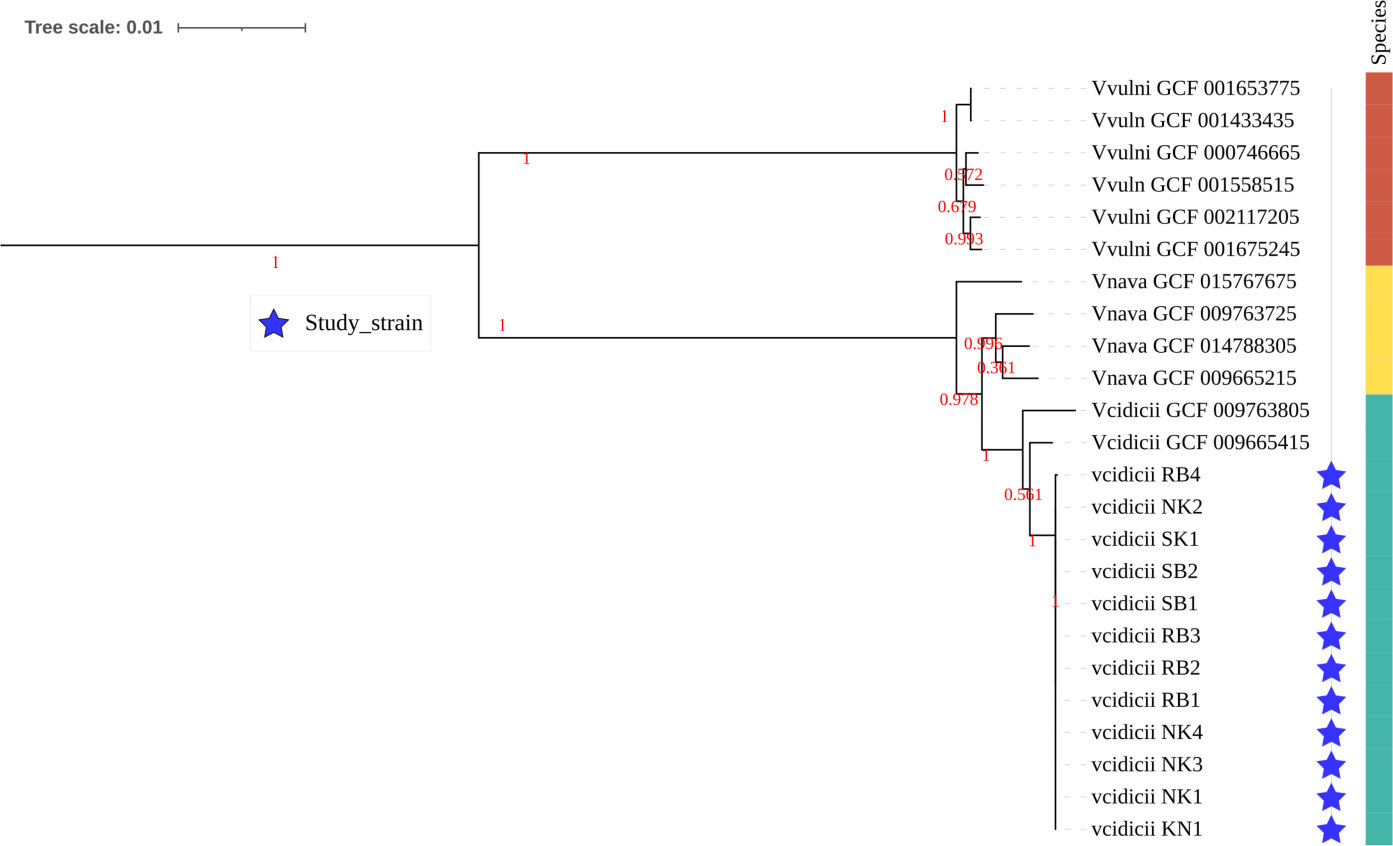
Comparative phylogenomic analysis of the 12 identified *V. cidicii* strains against complete public genomes of *V. cidicii*, *V. vulnificus*, and *V. navarrensis*. Single-copy marker gene phylogeny illustrating the clonality of our *V. cidicii* genomes and their close relatedness to the public *V. cidicii* genomes. *V. navarrensis* is the closest relative, followed by *V. vulnificus* (forming a separate clade). Abbreviations: Vnava for *V. navarrensis* and Vvulni for *V. vulnificus*. The tree was constructed using the maximum likelihood method with the Jones-Taylor-Thornton model (JTT) for amino acid substitution, with branch bootstrap values (SH-like support values of 0–1) calculated with 1,000 replicates for the reliability of each branch and displayed as red text on the tree in Fig. 1. The scale bar at the top of the tree indicates substitutions per site, with 0.01 corresponding to one substitution per 100 amino acid substitution sites.

The isolates were grown on Luria agar (Oxoid, Basingstoke, United Kingdom), and DNA was extracted using the Promega Maxwell automated DNA extraction system following the manufacturer’s protocol. Libraries for whole-genome sequencing (WGS) were prepared using the Nextera XT index kitv2 on an Illumina MiSeq platform for paired-end sequencing with a read length of 151. Sequencing yielded total read counts from 303,510 to 1,205,302, with total base counts between 64,202,136 and 27,603,6192 (Table 1). All bioinformatic packages used for analysis were run with default parameters. Duplicates, adapters, and sequence filtration were performed using Fastp (v.0.23.2) (5). Genome assembly was performed with SPAdes (v3.15.5) (6), and the quality of the assemblies was assessed with Quast (3.0.2) (7, 8). Each genome contained about 200 contigs, an N50 of about 130 kb, a final coverage determined with qualimap (v.2.3) (9) of 30×–95× and a size of 4.7 Mb with a GC content of 47.8% (Table 1). Species identification was performed using a dual sequence-based approach with Kraken (v.2.1.2) (10) and GTDB-Tk (v2.4.0) (11, 12), which confirmed the isolates as *V. cidicii*. We used GtoTree (v.1.8.8) (13) for comparative phylogenetic analysis of our *V. cidicii* against complete public genomes of *V. cidicii* and related species (*Vibrio navarrensis* and *V. vulnificus*). GToTree’s automated pipeline identified conserved single-copy marker genes from Gammaproteobacteria in the genomes, which were aligned, and the phylogeny was inferred using FastTree (v2.1.11) (14). This analysis reveals that our *V. cidicii* was clonal (Fig. 1) and remained closely related to the *V. cidicii* genomes from RefSeq. We also confirm that their closest related species is *V. navarrensis,* and *V. cidicii* is distant from *V. vulnificus* that is shown on a separate clade (Fig. 1).

**TABLE 1:** Summary of sequencing reads and assembly features of 12 *V. cidicii* strains

ID	Sampling date and sea temperature (°C)	Sample features		Read features				Assembly features				Final coverage	Reads accession	Assembly accession
				Read 1		Read 2								
		Geographical origin and GPS coordinates	Sample type	Total reads	Total bases	Total reads	Total bases	Contigs (bp)	Total length (bp)	GC (%)	N50			
KN1	03 August 2023; 22	Køge Nord beach: 55.499762N; 12.172863E	Green algae	443,300	105,012,242	443,300	90,809,789	184	4,695,634	47.8	128,131	41	ERR13699357	GCA_964276255
NK1	07 August 2023; 20	Nykøbing: 54.76906N; 11.87425E	Water	505,187	120,232,650	505,187	102,114,931	219	4,724,187	47.8	132,497	46	ERR13699358	GCA_964276245
NK2	07 August 2023; 20	Nykøbing: 54.76906N; 11.87425E	Water	463,445	109,775,011	463,445	94,628,717	221	4,727,428	47.8	131,783	42	ERR13699359	GCA_964276355
NK3	07 August 2023; 20	Nykøbing: 54.76906N; 11.87425E	Water	513,736	120,897,433	513,736	102,780,660	227	4,725,332	47.8	128,412	46	ERR13699360	GCA_964276285
NK4	07 August 2023; 20	Nykøbing: 54.76906N; 11.87425E	Water	478,811	113,296,671	478,811	99,143,354	207	4,719,089	47.8	128,181	44	ERR13699361	GCA_964276305
RB1	15 August 2023; 21.5	Rødby Bredfjed: 54.66134N; 11.35447E	Water	303,510	76,341,648	303,510	64,202,136	225	4,724,203	47.8	131,676	30	ERR13699362	GCA_964276345
RB2	15 August 2023; 21.5	Rødby Bredfjed: 54.66134N; 11.35447E	Water	368,072	90,825,708	368,072	77,636,849	230	4,716,403	47.8	128,147	35	ERR13699363	GCA_964276275
RB3	15 August 2023; 21.5	Rødby Bredfjed: 54.66134N; 11.35447E	Water	991,262	243,265,115	991,262	211,890,625	178	4,730,068	47.8	165,406	94	ERR13699364	GCA_964276335
RB4	15 August 2023; 21.5	Rødby Bredfjed: 54.66134N; 11.35447E	Water	1,205,302	276,036,192	1,205,302	248,116,127	221	4,830,518	48.0	2955	95	ERR13699365	GCA_964276315
SB1	10 August 2023; 20.5	Solrød beach: 55.53194N; 12.21944E	Water	530,511	131,912,537	530,511	110,371,897	221	4,729,996	47.8	137,261	50	ERR13699366	GCA_964276265
SB2	10 August 2023; 20.5	Solrød beach: 55.53194N; 12.21944E	Water	882,711	220,422,061	882,711	188,291,996	173	4,721,413	47.8	165,406	85	ERR13699367	GCA_964276295
SK1	05 August 2023; 18	Skagen: 57.7148N; 10.5983E	Water	363,985	89,799,826	363,985	75,541,051	188	4,686,900	47.8	155,473	35	ERR13699368	GCA_964276325

The 12 genomes have no antimicrobial resistance genes, no plasmids, and, so far, no multi locus sequence typing (MLST) scheme. The immunoreactive lipoprotein gene *Ilp*A, previously reported in *V. vulnificus* playing a role in evading the host immune system (15), was the only virulence-associated marker found. This paper highlights the importance of WGS analysis in the accurate classification of emerging pathogens.

## Data Availability

All the raw reads and the assemblies of the 12 *Vibrio cidicii* genomes reported in this study have been submitted to the European Nucleotide Archives and are publicly accessible under the project accession number PRJEB80374, with detailed accession numbers to each reads collection and assemblies available in Table 1.
